# Local tumour hyperthermia as immunotherapy for metastatic cancer

**DOI:** 10.3109/02656736.2014.968640

**Published:** 2014-11-28

**Authors:** Seiko Toraya-Brown, Steven Fiering

**Affiliations:** ^a^ Department of Microbiology and Immunology, Geisel School of Medicine at Dartmouth HanoverNew Hampshire; ^b^ Department of Genetics, Geisel School of Medicine at Dartmouth HanoverNew Hampshire, and; ^c^ Norris Cotton Cancer Center Lebanon, New HampshireUSA

**Keywords:** Biology, immunotherapy, local hyperthermia, tumor immunology, tumor immunotherapy

## Abstract

Local tumour hyperthermia for cancer treatment is currently used either for ablation purposes as an alternative to surgery or less frequently, in combination with chemotherapy and/or radiation therapy to enhance the effects of those traditional therapies. As it has become apparent that activating the immune system is crucial to successfully treat metastatic cancer, the potential of boosting anti-tumour immunity by heating tumours has become a growing area of cancer research. After reviewing the history of hyperthermia therapy for cancer and introducing methods for inducing local hyperthermia, this review describes different mechanisms by which heating tumours can elicit anti-tumour immune responses, including tumour cell damage, tumour surface molecule changes, heat shock proteins, exosomes, direct effects on immune cells, and changes in the tumour vasculature. We then go over *in vivo* studies that provide promising results showing that local hyperthermia therapy indeed activates various systemic anti-tumour immune responses that slow growth of untreated tumours. Finally, future research questions that will help bring the use of local hyperthermia as systemic immunotherapy closer to clinical application are discussed.

## Hyperthermia therapy for cancer treatment

Hyperthermia therapy is a treatment approach in which the temperature of a particular area of the body or the whole body is heated above normal temperatures to achieve therapeutic effects. There are three basic hyperthermia categories, local, regional and whole body. When local hyperthermia is used for cancer treatment, heat is applied to a solid tumour. The heating temperature can be as high as 80 °C when the purpose is to completely ablate the tumour [1], or can be in the range of 41–45 °C when aiming for certain physiological effects including cell death without causing serious injury to adjoining normal tissues [2]. Alternatively, the temperature of 39–41 °C can be used to mimic fever-range effects that do not damage any tissues. Regional hyperthermia is applied to a relatively large area, such as the peritoneal cavity or a limb. Whole-body hyperthermia is used in conjunction with other therapies to treat metastatic cancer that has spread throughout the body by systemic heating, and usually the fever-range temperature of 39–41 °C is used [3].

Currently most cancer patients die not from the primary tumour, but from metastatic disease, and the only approach to treating unrecognised metastases is chemotherapy. Immune-based cancer therapies are on the cusp of becoming part of the standard of care for many cancers because the immune system can actively seek and eliminate occult metastatic disease that may often survive chemotherapy. Recent studies indicate that many cytotoxic chemotherapeutic drugs mediate their effects in part by stimulating anti-tumour immunity [4–7]. With the increasing appreciation of the important role of the immune system in cancer treatment, there is growing interest in the potential of local tumour hyperthermia to activate anti-tumour immune responses.

## History of hyperthermia therapy for cancer

The use of heat for cancer treatment goes back to Ancient Egypt. According to the Edwin Smith Papyrus, a record of scientific approaches to medicine in Ancient Egypt, an Egyptian polymath named Imhotep burned off masses growing on the breast with a heated poker around 2600 BC. Certainly by 2000 BC, local destruction of tumours using cautery had become a widely used method for cancer therapy [8]. In Ancient Greece, Hippocrates stated in his Aphorism 87, ‘Those diseases that medicines do not cure are cured by the knife. Those that the knife does not cure are cured by fire. Those that fire does not cure must be considered incurable’ [8]. Although understanding of the immune system was primitive, in the late 19th century, physicians started realising that heat can do more than just kill tumours.

It had also been historically noted that infections were occasionally associated with cancer remission. In 1866 Busch in Germany reported that a malignant sarcoma on his patient underwent complete remission within two years after experiencing erysipelas infection [8]. William Coley, a surgeon in New York, decided to further investigate this phenomenon and, starting in the 1890s, he treated almost 900 inoperable cancer patients with various bacterial extracts named Coley’s toxins. Indeed, he achieved over 60% regression rate and over 20% cure rate [9], and this large-scale trial is famous as the first documented cancer immunotherapy study.

Since Coley’s patients developed fever that persisted throughout the treatment [9], the potential of fever in treating cancer was also recognised by Coley. In fact the 5-year survival rate was later found to be directly related to the achieved body temperatures [10,11]; 60% of the patients who experienced fever above 38.5 °C survived after 5 years, while only 28% if the fever remained below 38.5 °C. It is unclear whether the better effects with higher fever were from the fever itself or because stronger immune responses correlate with higher fevers. Despite the inability to distinguish effects of fever from effects of infection, Coley’s results at least generated scientific interest in raising body temperatures, not just by fever but also by systemic hyperthermia, which is physiologically different from fever, for inducing anti-cancer effects. It is intriguing that both cancer immunotherapy and modern hyperthermia therapy for cancer originate in the same studies by Coley, which stimulated the eventual finding that the efficacy of hyperthermia therapy beyond local ablation relies on immune activation [12].

In 1913, William Mayo documented some directly relevant clinical observations concerning the impact of local tumour hyperthermia [13]. He found less tumour dissemination post-surgery and an increase in the cure rate when he heated cervical tumours with a cautery before vaginal hysterectomy. Importantly, he observed ‘little if any difference in the ultimate results’ unless he gave enough time between local hyperthermia and surgical removal. Although the report does not describe treatment details such as heating temperatures or duration, it tells us that his improved outcome was not simply because the tumour cells were killed in a more thorough manner by adding heat. Rather, there was a protective mechanism that required time to develop before surgical resection, which we now assume was most likely activation of anti-tumour immunity.

Despite Mayo’s inspiring finding, the potential of locally heating tumours before surgery in order to stimulate anti-tumour immune responses had no further reports until recently. While there are multiple reasons for this, it is at least partly due to lack of technologies to maintain tumours at a specific temperature in a uniform manner for a sustained time period and to measure temperatures precisely during the treatment [8]. Currently, clinical local hyperthermia is used either for ablation purposes as an alternative to surgery or in combination with chemotherapy and/or radiation therapy to enhance the tumouricidal effects of those therapies [2], but not for the specific purpose of boosting anti-tumour immunity against known or occult metastatic disease.

## Methods for inducing local hyperthermia

There are several methods for inducing local hyperthermia. One historical method is to expose tumours to external lights such as an infrared light or to submerge the tumour site in a water bath. These strategies are suitable for tumours that are easily accessible, for example skin tumours, but not for tumours on internal organs, and even for skin tumours precise temperature and spatial control is difficult. An actively utilised method is to insert metallic probes into the tumour and deliver energies that preferentially deposit heat in the metal probe, such as microwaves or radiowaves, to raise the tumour temperature. This method enables rapid and large increases in the temperature and is therefore good for ablation purposes. However, it tends to form a significant temperature gradient across the tumour [14] and this large temperature gradient makes it less attractive for treatments that aim for immunostimulatory responses that are reported to be sensitive to small temperature differences [15,16]. Recent progress in high intensity focused ultrasound (HIFU) techniques have enabled non-invasive heating of internal organs and show promise for local hyperthermia of tumours not near the surface [17]. Imaging techniques are often used to make sure that the heating is occurring at intended locations and temperature ranges within the tumour [18]. Recently, nanomaterials with appropriate external energy sources to activate the nanomaterials to produce heat are being used for local hyperthermia [19]. When the nanoparticles are reasonably uniformly distributed throughout the tumour, this method enables more precise temperature control and more uniform temperature distribution within the tumour [20]. This improved distribution and control of the heat is facilitating studies of how local hyperthermia of tumours can stimulate systemic anti-tumour immune responses.

## Mechanisms by which local hyperthermia induces anti-tumour immune response

Temperatures in the range of 39–45 °C can arrest cell proliferation and kill cells. The effects are dependent on a combination of temperature and time of exposure to that temperature that together are referred to as thermal dose [21]. When cells are exposed to elevated temperature, several changes occur. Heat alters membrane characteristics, leading to modification in cell morphology, intracellular sodium and calcium levels and membrane potential [22–25]. Surprisingly, none of these phenomena correlates well with the cell death rate and therefore do not seem to be the direct mechanism of heat-induced cytotoxicity [22–25]. Other than membranes, secondary structures of proteins are thought to be the most sensitive biomolecules to heat and protein denaturation likely mediates many of the effects of mild hyperthermia [21]. Although DNA itself is not damaged at temperatures of 39–45 °C, *de novo* synthesis and polymerisation of DNA are more temperature sensitive due to denaturation and aggregation of synthetases and polymerases [26–29], and this is thought to greatly contribute to cell cycle arrest and cell death. Impaired functions of proteins responsible for other essential cell activities, such as DNA repair, are likely also involved. In general, following a sufficient thermal dose cells die through either necrosis, in which the cell rapidly loses membrane integrity, or apoptosis, in which programmed cell death is triggered, and each of these deaths has different immune modulatory activities [30].

**Figure 1.**
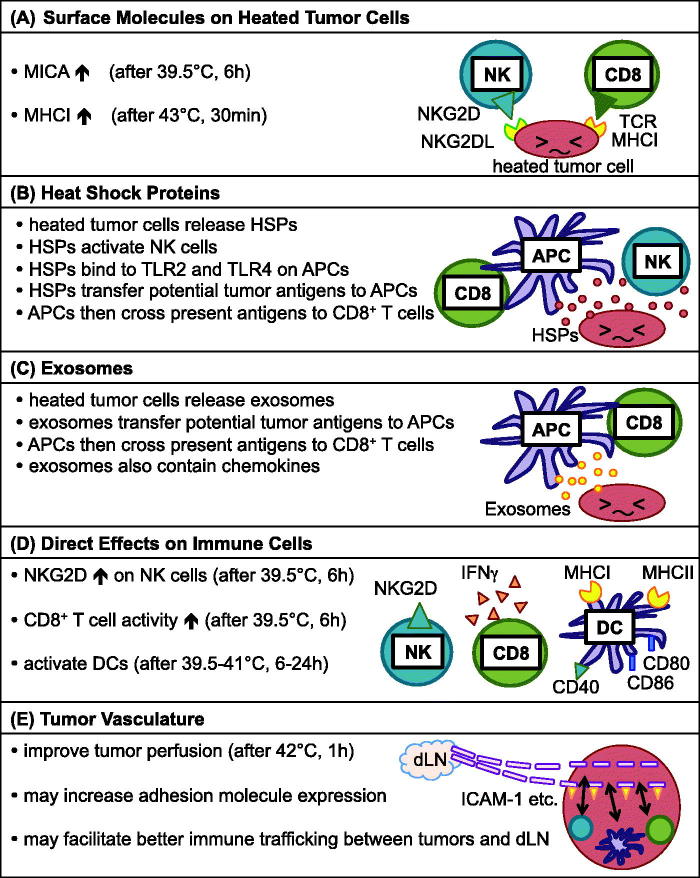
Different mechanisms of immune activation induced by locally heating tumours. (A) Heated tumour cells increase the surface expression of MICA, a NKG2D ligand, and MHC class I, making the tumour cells more sensitive to lysis by NK cells and CD8^+^ T cells, respectively. (B) Heated tumour cells release HSPs, which activate NK cells and APCs. HSPs contain potential tumour antigens, and APCs take up the HSP-antigen complex and cross present the antigen to CD8^+^ T cells. (C) Heated tumour cells release exosomes. Exosomes also contain potential tumour antigens, and APCs take up the antigen and cross present the antigen to CD8^+^ T cells. (D) Immune cells, such as NK cells, CD8^+^ T cells and DCs, in the tumour also get heated and become activated. (E) The tumour vasculature becomes more permeable and may have increased adhesion molecule expression after heating, which may facilitate better trafficking of immune cells between the tumour and dLN.

Originally, the purpose of local hyperthermia therapy was simply to burn off cancer cells to get rid of them, similar to that of surgery. Since the positive correlation between the thermal dose and cytotoxicity is known historically [31,32], it was thought the higher the heating temperature, the better. However, within the past 20 years increasing evidence shows that heating tumours at the temperature of 39–45 °C provides unexpected benefit, improvement of anti-tumour immunity [12]. Different mechanisms of immune activation occur at different temperatures within the mentioned range (Figure 1). One important concept in understanding the mechanisms by which local tumour hyperthermia can induce systemic anti-tumour immune responses is that studies on specific tumour lines may not be fully generalisable to all tumours but do indicate potential immune stimulatory changes to the tumour cells themselves.

## Surface molecules on heated tumour cells

Hyperthermia can increase the visibility of tumour cells to the immune system. Repasky’s group showed that heating tumour cells *in vitro* at 39.5 °C for 6 h increased surface expression of MICA, an NKG2D ligand, but not MHC class I [33], making the cells more sensitive to lysis by natural killer (NK) cells [33]. Kobayashi’s group showed that tumour cells heated *in vitro* at 43 °C for 30 min had increased surface MHC class I levels [34], which allows better recognition by CD8^+^ T cells. Increased lysis of tumour cells by NK cells and CD8^+^ T cells within the heated tumour can further improve anti-tumour immune responses, for instance by creating a more inflammatory cytokine milieu.

### Heat shock proteins

The topic that has been studied most extensively in the context of hyperthermia-induced anti-tumour immunity is the role of heat shock proteins (HSPs). HSPs are a heterogeneous group of molecular chaperones with various functions that are up-regulated when cells are stressed in a variety of manners, including heat exposure [35]. HSPs are usually divided into subgroups based on the molecular size; small HSP (<40 kDa), Hsp40, Hsp60, Hsp70, Hsp90 and Hsp100-110, among which Hsp70 is most recognised to be immunostimulatory. Hsp70 has an epitope that is recognised by NK cells and stimulates NK cell proliferation and cytolytic activities [36–39]. Hsp70 is also released by heat-stressed cells and directly binds to TLR2 and TLR4 on antigen-presenting cells (APCs) such as dendritic cells (DCs) to activate cytokine production and antigen uptake by the APCs [40–43]. Because HSPs are chaperones, HSPs released into the extracellular environment are often bound to proteins from within tumour cells, and therefore by being engulfed by APCs HSPs can transfer potential tumour antigens to APCs [42–44]. Srivastava’s group and others beautifully showed that those APCs are able to cross-present tumour antigens from HSP complexes to CD8^+^ T cells via MHC class I and thus elicit tumour-specific CD8^+^ T cell responses [44–46]. By prophylactically immunising mice with tumour-derived HSP complexes in a CD8^+^ T cell-dependent manner [47], this antigen cross-presentation pathway was utilised to generate anti-tumour immune responses that retarded tumour growth. HSPs are increased by heat or other stresses but this response takes time to develop, and it is possible that one mechanism by which mild hyperthermia is more stimulatory than higher levels of heat is that rapid necrosis kills cells before they can manifest increased HSP expression. However, milder heat that generates cells that are damaged and struggling to survive would increase HSP expression and therefore increased antigen cross-presentation through the pathway outlined above.

While many HSPs are considered generally immunostimulatory [40,41,43,48,49] and hence would help suppress tumour growth, negative roles of some HSPs in suppressing tumour growth are also reported [50–53]. Hsp90, for example, blocks apoptosis by directly interacting with and repressing the tumour suppressor protein p53 [51]. Although Hsp110-tumour antigen complex can stimulate DCs to produce inflammatory cytokines and prime antigen-specific naïve T cells, binding of Hsp110 to scavenger receptor A expressed on DCs reduces those activities by DCs [53]. Since HSPs are not only danger signals but also are lifeguards of the cell that protect cells against environmental stress [35], it is not surprising that some HSPs activate the immune system to attack the heated tumour cells while other HSPs prevent the heated cells from dying from the heat itself or from overly activated immune attack. Therefore, when applying *in situ* local hyperthermia, it should be noted that the overall outcome is influenced by the sum of all the effects by different HSPs. The amount of HSP production is dependent on the HSP subgroup, heating temperature and cell type. In the case of Hsp70 in the B16F10 mouse melanoma cell line, for example, Hsp70 release starts at 41 °C, reaches maximum at 43 °C and is completely diminished at 45 °C [54]. As for heating length, 30 min is better at inducing Hsp70 than 60 or 120 min [54]. These parameters can be very critical in eliciting maximal HSP-based immune activation, particularly when functions of certain HSPs are desirable over others. This temperature sensitivity highlights the need to understand temperature effects across a relatively narrow range of thermal doses and be able to control that thermal dose accurately and apply it uniformly to the tumour.

Besides heat, many kinds of physiological and chemical stress induce HSP expression. One of the most striking findings in recent cancer therapy research is that the efficacy of many chemotherapy drugs that have been used historically to kill cancer cells was actually relying on immune activation by chemotherapy-induced immunogenic cell stress and death [4–7]. In fact, tumour cells treated with those chemotherapy drugs release HSPs and those treated tumour cells activate DCs and T cells [6,7]. Therefore, it is not surprising if local tumour hyperthermia also works as an immunotherapy by inducing HSPs that stimulates anti-tumour immune responses. Furthermore, while HSP induction by local tumour hyperthermia is largely tumour-specific but only in the heated tumour, HSP induction by systemically administered chemotherapeutics is non-specific but also occurring in tumours of any location including unidentified tumours. This suggests that combination of local hyperthermia with certain chemotherapeutics may be synergistic in stimulating systemic anti-tumour immune responses. Cooperative benefits are often obtained in various combinations of immunotherapy and chemotherapy [55–57].

### Exosomes

Exosomes, small membrane vesicles of 30–100 nm in diameter, are normally released by cells and have roles in intercellular communication [58]. Tumour-cell derived exosomes contain enriched amounts of tumour antigens [59–62] and therefore are now recognised as potential immunostimulatory factors. Studies show that pulsing DCs with tumour-derived exosomes results in transfer of tumour antigens to DCs and those DCs stimulate tumour antigen-specific CD8^+^ T cell responses in mice [59] and human *ex vivo* systems [60,61].

Tumour cells experiencing stress, such as hypoxia and heat, also release increased amounts of exosomes [63–66]. The stress conditions are generally reflected in the content of exosomes [64] and hence also likely in the effects of the exosomes. Exosomes from heated tumour cells do seem to have increased ability to stimulate anti-tumour immune responses. Cao’s group showed that exosomes harvested from various human tumour cells heated at 42–43 °C for 1 h carry tumour antigens and act as an antigen source for APCs, inducing DC activation and tumour-specific CD8^+^ T cell responses in human HLA transgenic mice [65]. Chemokines such as CCL2, CCL3, CCL4, CCL5 and CCL20 are also contained in those exosomes and help attract DCs and both CD4^+^ and CD8^+^ T cells [66].

On the other hand, immunosuppressive properties of tumour-derived exosomes are also known. For example, some exosomes of tumour origin express death ligands such as FasL and TRAIL, triggering apoptosis of activated T cells [67,68]. Exosomes can contain NKG2D ligands and inhibit NKG2D-dependent cytotoxicity of NK cells and CD8^+^ T cells by blocking the NKG2D receptor [69]. There are also exosomes that induce the differentiation of and support functions of myeloid-derived suppressor cells and regulatory T cells through a TGF-β dependent mechanism [62,70]. Although these negative roles of tumour-derived exosomes in immune stimulation are not reported in the context of heat stress, the question of whether exosomes induced by locally heating tumours stimulate or suppress the anti-tumour immune response in total needs to be assessed carefully. Likely this balance will again depend on details of the specific tumour, the thermal dose and the existing immune response prior to treatment.

### Direct effects on immune cells

When tumours are treated with local hyperthermia, tumour-infiltrating immune cells will also be exposed to heat. Fever is a natural immune stimulatory mechanism associated with fighting infections, so it is not surprising that fever level hyperthermia has stimulatory effects on leukocytes. According to *in vitro* studies by Repasky’s group, heating human NK cells at 39.5 °C for 6 h does not alter the surface expression level of NKG2D but results in NKG2D clustering as seen on NK cells activated with IL-2, leading to better lysis activity [33]. They also showed that heating antigen-specific CD8^+^ T cells *in vitro* at 39.5 °C for 6 h enhances antigen-specific IFN-γ production and target tumour cell killing ability [71]. Culturing bone marrow-derived DCs at 39.5–41 °C for 6–24 h activates DCs as seen by up-regulation of MHC class I and II, CD40 and co-stimulation markers CD80 and CD86, and by better induction of antigen-specific T cell proliferation [72,73]. Activation of macrophages upon *in vitro* culture at 39.5–40 °C for 2–3 h is also reported [74–76]. Almost all relevant literature agrees that activation of immune cells by direct heating is only observed in fever-range temperatures but not at higher temperatures above 41 °C [77], highlighting the importance of selecting appropriate heating temperatures to suit the purpose.

### Tumour vasculature

Lastly, changes in the vasculature within the tumour may help immune cell mobilisation. *In situ* local hyperthermia increases the permeability of tumour vasculature. Dewhirst’s group showed that heating rat tumours at 42 °C for 1 h using a water bath increases the diameter of arterioles entering tumours by 35%, resulting in better tumour perfusion [78]. Others show better perfusion in an indirect manner, through increase in pO_2_ [79] or better delivery of therapeutic materials [80], but overall there is agreement that blood flow in the tumour is improved upon local hyperthermia in the range of 40–43 °C and that the effect is abrogated at temperatures higher than 43 °C due to haemorrhage. Since better perfusion has the potential to facilitate increased trafficking of immune cells including DCs and T cells between tumours and dLN, heating to temperatures of 40–43 °C may add further immune-mediated benefit.

Immune cell trafficking may be further improved through changes in the vasculature adhesion molecules. Evans’ group showed that whole body hyperthermia of 39.5 °C for 5 h in mice increased intratumoural IL-6 trans-signalling [81]. This increases ICAM-1 expression on the tumour vasculature and tumour-specific CD8^+^ effector/memory T cell trafficking into the tumour, both of which are recapitulated by intravenously administering IL-6 instead of giving whole body hyperthermia. Our lab has shown that locally heating tumours at 43 °C for 30 min increases the intratumoural concentration of IL-6 [16]. Therefore, it is possible that this local tumour hyperthermia treatment also increases ICAM-1 on the tumour vasculature and enhances trafficking of T cells or other immune cells into the tumour.

## Efficacy of local hyperthermia therapy and immune involvement

If the impact of local tumour hyperthermia is not stimulation of systemic immune response, then ablative thermal doses applied to the local tumour would be preferable to milder doses since it would eliminate the local tumour completely. With all the evidence that locally heating tumours would improve local anti-tumour immunity, it is important to determine whether the heat induces sufficient systemic immune responses to reject or at least retard the growth of untreated tumours. In fact, a variety of animal studies demonstrate the ability of hyperthermia of one tumour to affect the growth of other tumours not exposed to heat. Most researchers utilise subcutaneous or dermal tumour models for experimental feasibility. While many successful studies heat tumours at 42–45 °C, some studies show hyperthermia at ablation temperatures is also effective in eliciting anti-tumour immunity [1]. The most immunostimulatory thermal dose parameters are not fully determined yet and this is further exacerbated by limited information regarding temperature gradients across tumours during treatment. Once again, it would not be surprising if the optimal thermal dose differed for different tumour types.

### Hyperthermia at 42–45 °C

Groups led by Kobayashi, Honda and Jimbow, the pioneers of the field of nanomaterial-induced local hyperthermia for immunotherapy against cancer, apply an alternating magnetic field to induce heat production by nano-sized liposomes containing iron oxide that are directly injected into the tumour. Upon heating of T-9 rat glioma on one flank for 30 min (approximately 42–45 °C throughout the heating process), not only the heated tumour, but also the unheated T-9 tumour implanted on the contralateral flank of the same rat completely disappeared. This coincided with increased infiltration of NK cells, CD4^+^ T cells and CD8^+^ T cells in both the heated and unheated tumours [82], indicating possible roles of these lymphocytes.

They also showed that mice whose primary subcutaneous B16 melanoma was heated at 43 °C for 30 min have splenocytes with increased cytotoxicity specifically against B16 cells and show better resistance against secondary B16 rechallenge on the other flank compared to naïve mice [83]. Although the comparison was done with naïve mice, this at least implies that a single hyperthermia treatment is possibly capable of inducing anti-tumour efficacy, considering that B16 cells are in general poorly immunogenic. Furthermore, they found that DCs in the heated tumour migrate to the draining lymph node (dLN) better than DCs in the unheated tumour [84]. Interestingly, the rechallenge resistance was more efficiently induced when the heating was at 43 °C than at 41 °C or 46 °C [15], which means that the anti-tumour efficacy is sensitive to small temperature differences and an optimal temperature exists at least within this narrow range of heating temperatures.

Using solid iron oxide-based nanoparticles and an excisable dermal B16 tumour model, our lab compared mice whose primary tumours were heated and excised and mice whose primary tumours were unheated and excised [16]. Heating the primary tumour at 43 °C for 30 min induced increases in many cytokine and chemokines in the tumour, activated DCs in dLN, increased the frequency of and activated CD8^+^ T cells in dLN, and conferred resistance against rechallenge with B16 tumours given on both the primary tumour side and the contralateral side [16]. The resistance was not generated against unrelated Lewis Lung (LL) carcinoma, and hence the efficacy is specific to the kind of tumours heated, indicating a specific response against unique tumour antigens. Importantly, the same hyperthermia treatment did not induce resistance in mice depleted of CD8^+^ T cells, (but resistance was induced in mice that lacked either NK cells or IL-12), demonstrating that the anti-tumour efficacy is mediated by CD8^+^ T cells. Neither CD8^+^ T cell activation nor rechallenge resistance occurred when the primary tumour was heated at 45 °C instead of 43 °C, again suggesting the existence of an optimal temperature range for anti-tumour immune activation even within this narrow temperature range. This highlights the importance of precisely controlling the tumour temperature in a uniform manner, which can be done relatively easily in nanomaterial-mediated hyperthermia [85] as we also confirmed in our study [16].

Some studies using non-nanomaterial heating methods also produced valuable results. Kubes et al. heated subcutaneous B16 tumours at 42 °C for 7 min three times by applying microwaves [86]. This treatment increased CD8^+^ T cells and NK cells in the spleen, and splenocytes from treated mice showed higher cytotoxicity against B16 and NK-sensitive YAC-1 target cells than the untreated controls. Chen et al. heated subcutaneous Lewis Lung tumours at 42–43 °C for 1 h three times using microwaves [87]. They showed that Hsp70 produced by heated tumour cells activated tumour cells to produce chemokines that increased recruitment of DCs and CD4^+^ and CD8^+^ T cells into the tumour and that the recruitment was dependent on TLR4 expressed by tumour cells and DCs. Although these two studies did not show whether the immunological changes were sufficient to affect tumour growth of unheated tumours, the results at least tell us that heating tumours at 42–43 °C using microwave methods is also able to elicit anti-tumour immune responses.

### Hyperthermia at ablation temperatures

den Brok et al. transferred either splenocytes or serum from donor mice whose subcutaneous B16-ova tumours were heated twice for 80 sec by radiofrequency ablation (reached 75–80 °C) into naïve host mice [1]. When these host mice were then challenged with B16-ova, tumours grew less aggressively than when donor mice did not receive ablation treatment, showing that the immunological factor is sufficient to confer heat-induced resistance. Their results also teach us that optimal heating conditions exist in this high temperature range as well, although the use of the potent neo-antigen ovalbumin expressed by the tumour line limits the interpretation when applied to tumours that do not contain such a potent neo-antigen.

An interesting study by Bear et al. utilised gold nanoshells that generate heat upon exposure to near infrared radiation [88]. Heating subcutaneous B16 tumours for 3 min (temperature not mentioned, but high enough to ablate tumours) increased levels of inflammatory cytokines and chemokines in the serum, and induced maturation of DCs in dLN. However, this hyperthermia treatment also significantly promoted expansion of immunosuppressive myeloid-derived suppresser cells in the spleen, reduced T cell proliferation in the spleen, and failed to slow the growth of the unheated contralateral tumour. Interestingly, when adoptive transfer of B16-specific CD8^+^ T cells was combined with this hyperthermia therapy, the transferred CD8^+^ T cells expanded better in the spleen and dLN and the contralateral tumour grew slower than in adoptive transfer only mice. This is an encouraging example that shows hyperthermia therapy is able to cooperate with immunotherapy even when hyperthermia therapy itself is not potent enough to retard tumour growth.

## Conclusions and future questions

It is exciting to see the many promising results from the preclinical studies described above. To summarise, locally heating tumours at 39–45 °C can elicit anti-tumour immune responses (1) by enabling tumour cells to stimulate the immune system through increased surface expression of MICA or MHC class I and release of HSPs and/or exosomes, (2) by directly activating intra-tumoural immune cells such as NK cells, CD8^+^ T cells, and DCs, and (3) by improving immune-cell trafficking between the tumour and lymphoid organs. Local tumour hyperthermia at 42–45 °C in mice induces tumour-specific resistance against rechallenge in a CD8^+^ T cell-dependent manner. This efficacy is sensitive to small temperature differences, which means that there is a narrow optimal temperature range at least for a poorly immunogenic tumour. Since locally ablating tumours also generates splenocyte-mediated tumour specific resistance, an optimal temperature range may exist at higher temperatures and associated thermal doses as well. Thorough comparison of different temperatures/thermal doses is needed to fully understand what temperatures are most suitable for immune stimulation. Following identification of thermal dose optima, the underlying mechanisms that differentiate the responses to different thermal doses can be explored.

To make local hyperthermia therapy a more powerful immunological approach for clinical application, it is critical to optimise the treatment as much as possible. Hyperthermia treatment protocols in reported studies differ in numerous factors including not only heating temperatures but also heating methods and duration, treatment timing and intervals, cancer types and stages, and therapeutic readout. It is important to understand how each parameter influences immune activation and the therapeutic outcome and to rigorously compare various protocols with only one variable at a time. This will eventually enable adjustment of each parameter to maximise the treatment efficacy according to the individual clinical situation.

One of the questions needing further exploration is whether local hyperthermia cooperates with other clinically used therapies. It is likely that local hyperthermia therapy amplifies effects by other immunotherapies, such as checkpoint blockade and adoptive T cell therapy as already indicated by some studies [1,88]. Since heating tumours activates the immune system at least partly through increased and released HSPs [87] and some chemotherapy drugs also depend for their efficacy on HSPs [4,5,7], these two therapies may work additively, for example by enhancing tumour antigen presentation by APCs through increased total HSP release by tumour cells. Alternatively, one therapy may inhibit the other therapy’s effect, for example by modifying the type of HSP released or the proteins bound to HSP when they are released. An important variable of trials with human patients is how patients’ treatment history affects the efficacy of local hyperthermia therapy.

Further understanding the detailed biological and immunological mechanisms of how heating tumours *in situ* enhances anti-tumour immunity will help optimise the therapy for clinical application. Although there is still much room for improvement, emerging evidence demonstrates the potential of local hyperthermia therapy as a powerful tool to support other emerging approaches to cancer immunotherapy.

## Declaration of interest

Funding for this study came from a Dartmouth Center of Nanotechnology Excellence National Institutes of Health grant 1 U54 CA151662, a Center for Molecular, Cellular, and Translational Immunological Research National Institute of General Medical Sciences grant P20 RR15639, a Norris Cotton Cancer Support grant P30 CA023108, and Dartmouth Immunobiology of Myeloid and Lymphoid Cells grant 5T32AI007363-22. The authors alone are responsible for the content and writing of the paper.
